# The Use of Functional Chemical-Protein Associations to Identify Multi-Pathway Renoprotectants

**DOI:** 10.1371/journal.pone.0097906

**Published:** 2014-05-15

**Authors:** Jia Xu, Kexin Meng, Rui Zhang, He Yang, Chang Liao, Wenliang Zhu, Jundong Jiao

**Affiliations:** 1 Department of Nephrology, the Second Affiliated Hospital of Harbin Medical University, Harbin, China; 2 Department of Nephrology, the Fourth Affiliated Hospital of Harbin Medical University, Harbin, China; 3 Institute of Clinical Pharmacology, the Second Affiliated Hospital of Harbin Medical University, Harbin, China; 4 Institute of Nephrology, Harbin Medical University, Harbin, China; Semmelweis University, Hungary

## Abstract

Typically, most nephropathies can be categorized as complex human diseases in which the cumulative effect of multiple minor genes, combined with environmental and lifestyle factors, determines the disease phenotype. Thus, multi-target drugs would be more likely to facilitate comprehensive renoprotection than single-target agents. In this study, functional chemical-protein association analysis was performed to retrieve multi-target drugs of high pathway wideness from the STITCH 3.1 database. Pathway wideness of a drug evaluated the efficiency of regulation of Kyoto Encyclopedia of Genes and Genomes (KEGG) pathways in quantity. We identified nine experimentally validated renoprotectants that exerted remarkable impact on KEGG pathways by targeting a limited number of proteins. We selected curcumin as an illustrative compound to display the advantage of multi-pathway drugs on renoprotection. We compared curcumin with hemin, an agonist of heme oxygenase-1 (HO-1), which significantly affects only one KEGG pathway, porphyrin and chlorophyll metabolism (adjusted *p* = 1.5×10^−5^). At the same concentration (10 µM), both curcumin and hemin equivalently mitigated oxidative stress in H_2_O_2_-treated glomerular mesangial cells. The benefit of using hemin was derived from its agonistic effect on HO-1, providing relief from oxidative stress. Selective inhibition of HO-1 completely blocked the action of hemin but not that of curcumin, suggesting simultaneous multi-pathway intervention by curcumin. Curcumin also increased cellular autophagy levels, enhancing its protective effect; however, hemin had no effects. Based on the fact that the dysregulation of multiple pathways is implicated in the etiology of complex diseases, we proposed a feasible method for identifying multi-pathway drugs from compounds with validated targets. Our efforts will help identify multi-pathway agents capable of providing comprehensive protection against renal injuries.

## Introduction

Complex human diseases, also known as multi-gene diseases, are caused by the integration of the cumulative effects of genetic, environmental, and lifestyle factors. According to this definition, the majority of nephropathies could be described as complex. Recently, large-scale approaches such as gene microarray techniques have been applied to reveal the gene expression signatures of pathological renal cells and tissues [1], [2]. Numerous genes are implicated in renal pathology. Additionally, functional experiments have validated the co-existence of numerous aberrant pathways that are involved in inflammation, cell damage, renal fibrosis, and faulty repair functions [3]–[6]. These observations suggest that a multi-intervention strategy is the optimal approach to the maintenance of healthy renal function and the repair of damaged kidney tissue.

Abundant evidence has shown that most drugs are associated with multiple proteins rather than specifically targeting only one protein in a lock and key manner [7]. This provides a rational opportunity for pharmacological multi-intervention via multi-target drugs or their combinations [8]. Drugs that target multiple proteins have the potential to simultaneously regulate more than one disease-related pathway and provide more comprehensive therapy. Thus, we present a novel concept of multi-pathway drugs similar to the concept of multi-target drug previously proposed by Csermely et al [9]. We propose that a multi-pathway drug should be properly defined as a drug that can have a significant impact on multiple signaling pathways associated with diseases.

In the present study, we aimed to search for drugs with multi-pathway effects from STITCH 3.1, the world's largest database of well-defined and accepted chemical-protein associations [7]. With such a large collection of multi-target drugs, theoretically, a certain number of multi-pathway drugs should be found. Considering the significant relationship between the number of drug targets and the associated side effects [10], it was necessary to evaluate the quantitative effectiveness of a multi-target drug on pathway intervention. For this purpose, we proposed a parameter defined as pathway wideness (PW). We accomplished this by applying DAVID, a set of functional annotation tools, on revealing significantly over-represented pathway regulation exhibited by multi-target drugs [11]. Furthermore, the renoprotective potential of the drugs with a high PW (PW>0.6) was evaluated on the basis of previous experimental studies. An in vitro model of H_2_O_2_-treated glomerular mesangial cells was also used to examine the pluralistic effects of the multi-pathway drug curcumin. Curcumin displays high PW, in comparison with another renoprotectant, hemin that is a specific agonist of heme oxygenase-1 (HO-1). In summary, we demonstrate a method that allows a meaningful search for multi-pathway drugs with the potential of comprehensive protecting against renal injuries.

## Materials and Methods

### Chemical-protein associations and PW calculation

In this study, chemicals that are associated with multiple proteins (n≥10) were retrieved from the STITCH 3.1 database. A confidence score of 0.900 was selected as the threshold [7]. All chemical-protein associations were imported into Cytoscape [12] for data storage and topological analysis. The number of associated proteins was calculated for each chemical. Subsequently, each chemical was investigated to determine which signaling pathway with its associated proteins would be most significantly affected. This was accomplished by uploading their official gene symbol names onto the online functional annotation tool of DAVID [11]. Homo sapiens was selected as the species background and the “KEGG_pathway” annotation was chosen. Over-represented regulation on a Kyoto Encyclopedia of Genes and Genomes (KEGG) pathway [13] was considered statistically significant only if Benjamini-adjusted *p*<0.05 [14]. The parameter PW for each chemical was calculated as the number of significantly over-represented KEGG pathways affected, divided by the number of the associated proteins.
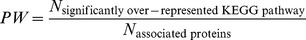



A high PW value indicates that a given chemical is efficient in intervening signaling pathways as much as possible by virtue of a limited number of targets. To investigate whether the affected pathways were nephropathy-related, the Gene Prospector online tool in HuGE Navigator was applied to find literature-reported nephropathy-related genes [15]. The online functional annotation tool of DAVID was then used to identify nephropathy-related pathways.

### Reagents, cell culture, and H_2_O_2_ treatment

Hemin, zinc protoporphyrin (ZnPP), and curcumin were purchased from Sigma-Aldrich Co. (USA). The anti-LC3 antibodies were obtained from Cell Signaling Technology, Inc. (USA). A stable human mesangial cell line (kindly donated by Dr. J. D. Sraer, Hopital Tenon, Paris, France) was established by transfection and immortalization by the viral oncogene large T-SV40 of human mesangial cells isolated from normal human glomeruli [16], and were cultured as described previously [17]. In brief, these cells were cultured in RPMI 1640 medium supplemented with 10% fetal bovine serum (Gibco, Grand Island, NY, USA). The cells between passages 3 and 15 were used and then sub-cultured in serum-deprived medium (1% FBS) for 12 h before use in subsequent experiments. The cells were treated with 125 µM H_2_O_2_ for 24 h.

### Cell viability assay

The 3-(4,5-dimethylthiazolyl-2)-2,5-diphenyltetrazolium bromide (MTT) assay was used to determine the cell viabilities based on the mitochondrial dehydrogenase activity. In brief, the cells were seeded in a 96-well plate at a density of 2.5×10^4^ cells/well and allowed to adhere to the wells for 24 h before H_2_O_2_ administration and drug treatments. The media from each well was then removed and replaced with PBS solution containing 0.5 mg/mL MTT, and the plates were incubated at 37°C for 4 h. The remaining supernatant was then removed. DMSO (150 µL) was added to each well and mixed thoroughly to dissolve the formazan crystals formed. After incubation for 10 min, the cell viability was determined by measuring the absorbance of each well at 490 nm.

### Caspase-3 activity assay

Caspase-3 activity was determined using a caspase-3 activity assay kit (Beyotime Institute of Biotechnology, China), which is based on the ability of caspase-3 to convert acetyl-Asp-Glu-Val-Asp *p*-nitroanilide to a yellow formazan product, *p*-nitroaniline. The detailed analysis procedure is described in the manufacturer's protocol (Beyotime Institute of Biotechnology, China). Caspase-3 activity, which was defined as the ratio of the *p*-nitroanilide content to the amount of total protein present, was determined by detecting the absorbance of *p*-nitroanilide at 405 nm by using a microliter plate reader (Bio-TEK Epoch, Bio Tek Instrument, VT, USA).

### Detection of reactive oxygen species (ROS)

ROS levels in the cells were quantified using an ROS assay kit (Beyotime Institute of Biotechnology, China). The cells were harvested by trypsin and exposed to 10 µM DCFH-DA for 20 min at 37°C. A fluorescence characteristic was then observed when DCFH-DA was intracellularly oxidized to DCF. The labeled cells were washed twice in PBS and then subjected to cytometric analysis at an excitation wavelength of 488 nm and an emission wavelength of 525 nm (BD FACSAria, USA). The ROS level was represented by the fluorescence intensity. The number of analyzed cells was 1×10^4^ in each individual experiment.

### Western blot

Cellular proteins were extracted with lysis buffer containing 1% protease inhibitor solution. The protein concentrations were subsequently determined using a BCA protein assay kit (Bio-Rad, USA). The proteins were then electrophoresed by 15% SDS-PAGE and transferred onto nitrocellulose membranes. The membranes were blocked with 5% non-fat dry milk in PBS for 2 h at room temperature and incubated with the primary antibodies at 4°C overnight. Afterward, the membranes were washed with PBS-0.1% Tween 20 and incubated with fluorescence-conjugated goat anti-rabbit IgG or goat anti-mouse IgG at a dilution ratio of 1∶10,000 (Invitrogen, USA) at room temperature for 1 h. The western blot bands were quantified using the Odyssey infrared imaging system (LI-COR, USA).

### Statistical analysis

All of the data are expressed as means ± SEM. The data analysis was performed using student's *t*-test or one-way ANOVA followed by Tukey's multiple comparison test. Differences were considered statistically significant only if *p*<0.05.

## Results

### Functional annotation analysis showed the existence of multi-pathway drugs

By applying the double restrictions of a confidence score of 0.9 and target numbers of 10, we identified 734 multi-target chemicals from the chemical-protein association database STITCH 3.1 (Table S1). The endogenous substance adenosine triphosphate was assigned with the maximum number of human proteins (n = 1330), followed by adenosine diphosphate that was associated with 1236 proteins and hydrogen ion that targets 1132 proteins. Curcumin has only 44 human protein partners, despite that it was considered to be involved in a wide range of pharmacological actions in addition to exhibiting strong antioxidant effects [18]–[21]. Moreover, some recognized specific agents, such as the PI3K-specific inhibitor wortmannin, have revealed 52 chemical-protein associations.

**Figure 1:**
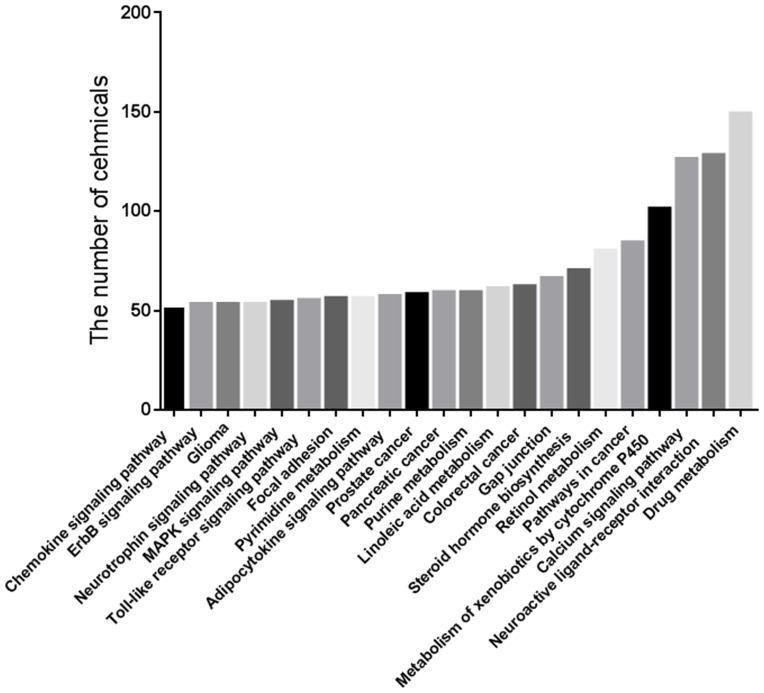
Most influenced KEGG pathways by chemicals.

**Figure 2:**
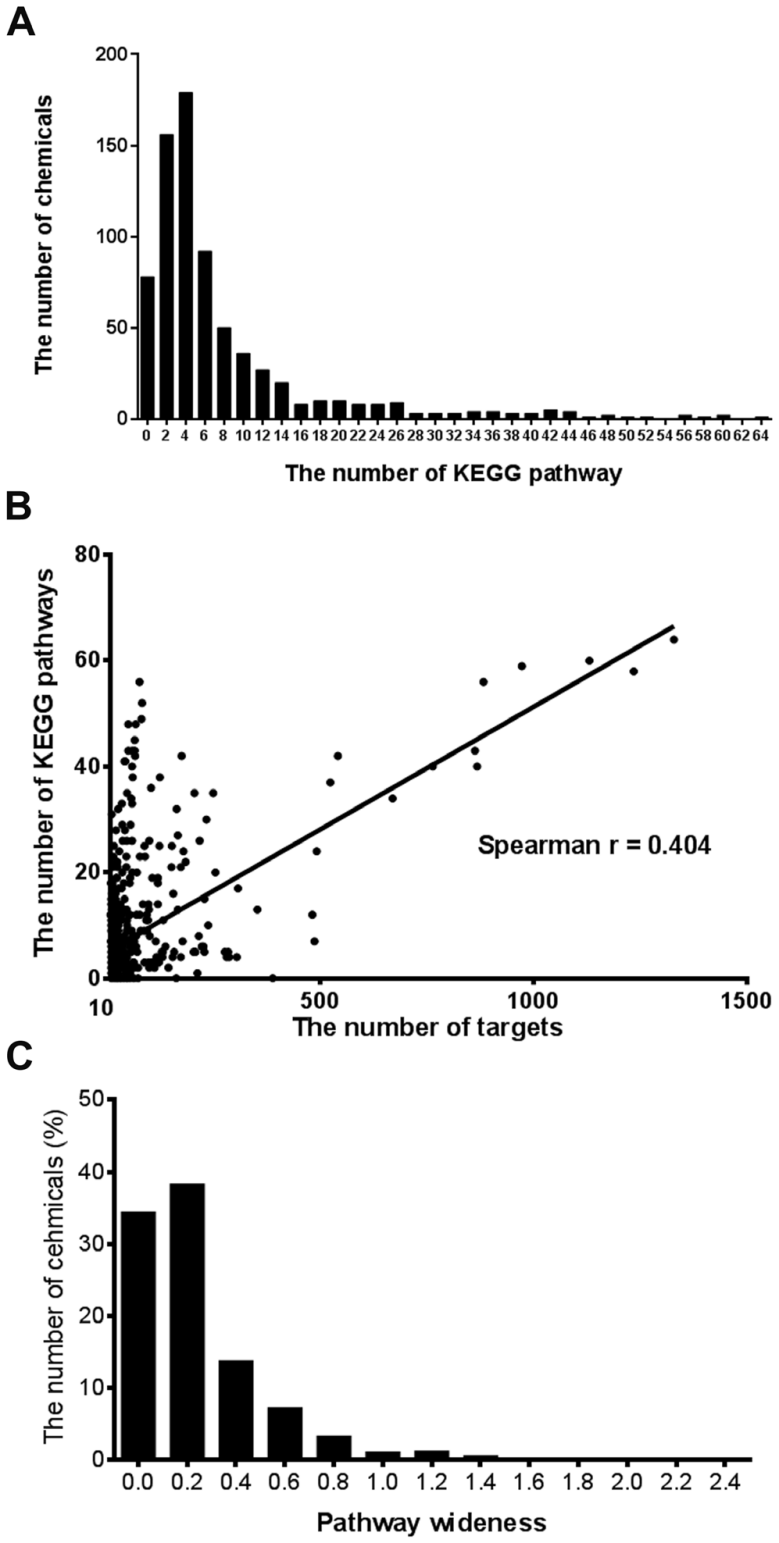
Analysis results of chemical and KEGG pathway associations. A. Distribution of chemicals according to the number of KEGG pathways. B. Correlation relationship between chemical targets and KEGG pathways in count. C. Distribution of chemicals according to pathway wideness.

We also investigated the regulatory potential of the 734 multi-target chemicals on KEGG pathways and found 5468 chemical-pathway associations (Table S2). Figure 1 shows that the pathways most affected were *drug metabolism* (149 chemicals), *neuroactive ligand-receptor interaction* (128 chemicals), and *calcium signaling pathway* (126 chemicals). Targeting multiple proteins does not mean that one drug can affect a remarkable intervention on multiple signaling pathways (Figure 2A). Seventy-eight chemicals failed to have significant impact on any KEGG pathway (Benjamini-adjusted *p*>0.05). However, 593 chemicals were identified as multi-pathway chemicals because they significantly regulated more than one KEGG pathways. Many of these are clinical drugs and natural active compounds (Table S1). There was diverse effectiveness in the ability of chemicals to intervene in pathways, and chemicals with more targets did not necessarily target more pathways. We observed a weak correlation between the number of targets and the number of KEGG pathways affected (Spearman r = 0.404, Figure 2B). By calculating the PW, we determined the most effective chemicals (Table S1 and Figure 2C). Among the 734 chemicals analyzed, 65 chemicals were found to effectively influence multiple KEGG pathways (PW>0.6). We called them high-PW chemicals.

### Pathway analysis showed multi-pathway renoprotectants

**Figure 3:**
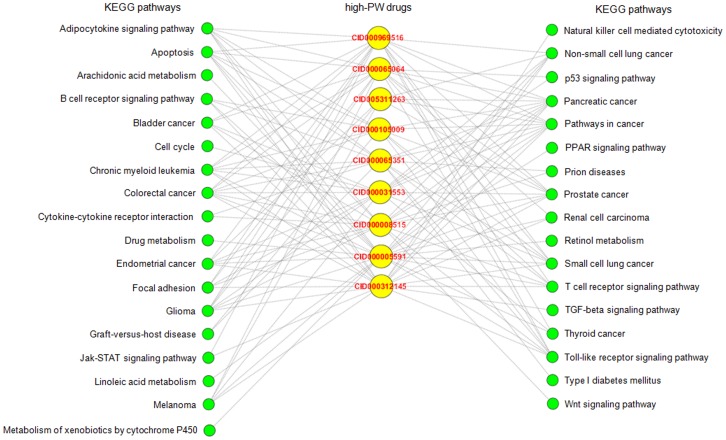
Influence on nephropathy-related KEGG pathways by high-PW drugs. CID000969516: curcumin; CID000065064: EGCG; CID005311263: lysophosphatidic acid; CID000105009: PDTC; CID000065351: pyrrolidine dithiocarbamate; CID000031553: silibinin; CID000008515: SP600125; CID000005591: troglitazone; CID000312145: wortmannin.

The Gene Prospector online tool identified 1841 nephropathy-related genes. The online functional annotation tool of DAVID identified 58 KEGG pathways, which are nephropathy-related (Table S3). Forty-two high-PW chemicals were found that each of them could influence less than three nephropathy-related pathways. This result implies that these chemicals are used as potential source of the multi-pathway renoprotectants. Furthermore, we identified nine multi-pathway renoprotectants of high PW by using the chemical name and renoprotectant as search terms in PubMed database (Figure 3 and Table S4). Among them, curcumin had been the most extensively studied (25 articles), followed by EGCG, an active ingredient in green tea (5 articles).

### In vitro comparison revealed the advantage of multi-pathway renoprotectants: a case study

**Figure 4:**
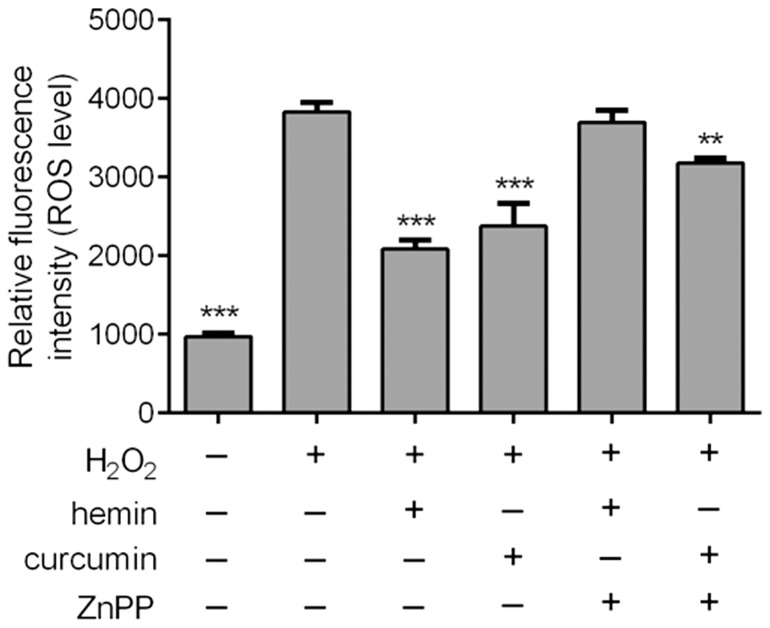
Effect of hemin, ZnPP and curcumin on the content of ROS in H_2_O_2_-treated mesangial cells. ** *p*<0.01 and *** *p*<0.001 compared with H_2_O_2_. n = 3.

**Figure 5:**
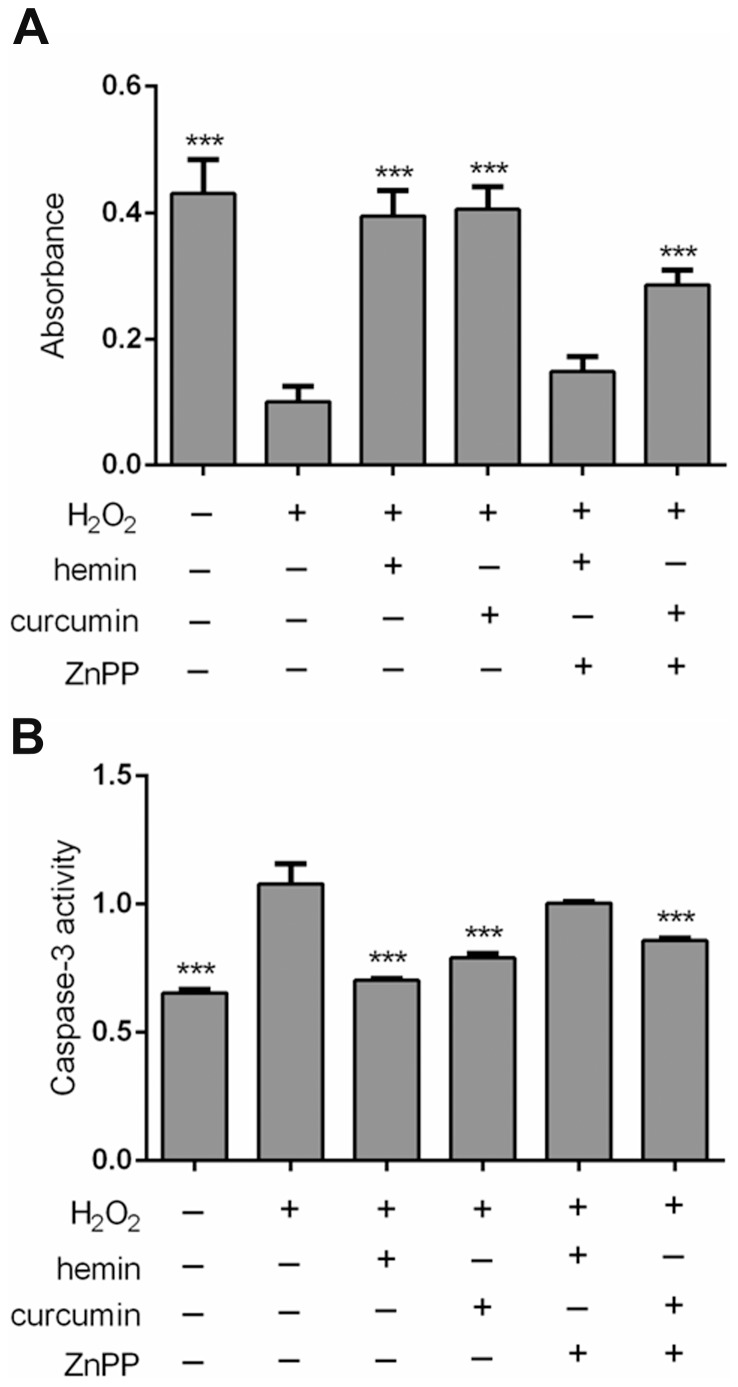
Effect of hemin, ZnPP and curcumin on cellular viability (A) and caspase-3 activity (B) in H_2_O_2_-treated mesangial cells. *** *p*<0.001 compared with H_2_O_2_. n = 6.

In this study, curcumin was selected as an illustrative chemical to demonstrate the advantage of simultaneous intervention on multiple signaling channels. In comparison, the single-pathway renoprotectant hemin was used as a reference drug. A previous study demonstrated hemin as a renoprotectant against ischemic renal injuries [22]. We confirmed that both curcumin and hemin, at the same concentration of 10 µM, could relieve oxidative stress induced in glomerular mesangial cells by H_2_O_2_ treatment (Figure 4). However, when the specific HO-1 inhibitor ZnPP was combined with curcumin or hemin, we observed that the oxygen radical scavenging activity of hemin was completely lost, whereas only a partial decline in the activity of curcumin was observed. Similar findings were noted in cell apoptosis assays (Figure 5). Taken together, this evidence suggests that curcumin actively intervenes in multiple pathways.

**Figure 6:**
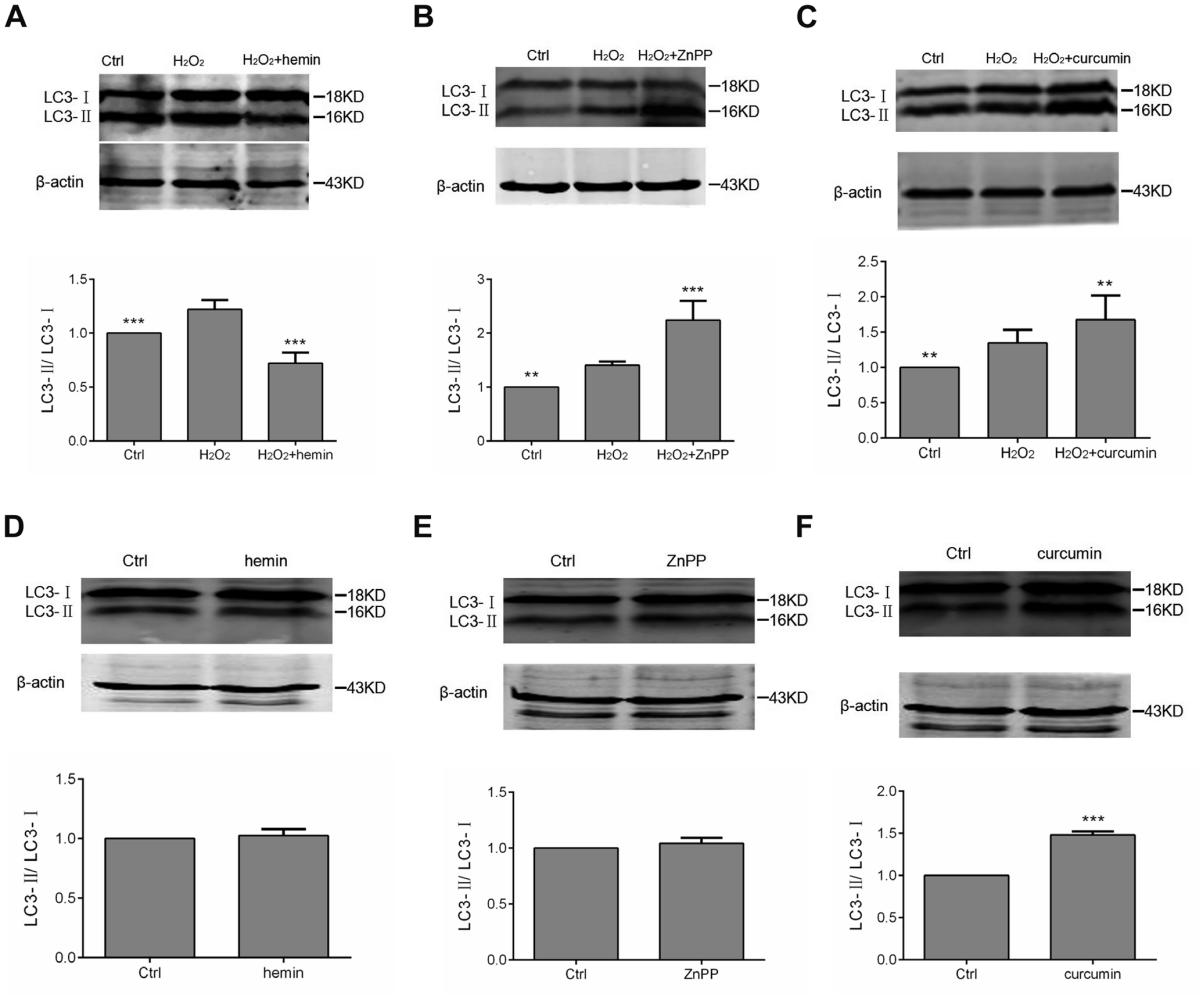
Effect of hemin, ZnPP and curcumin on autophagy of H_2_O_2_-treated mesangial cells (A–C) and normal mesangial cells (D–F), respectively. ** *p*<0.01 and *** *p*<0.001 compared with H_2_O_2_ or Ctrl. Ctrl: normal mesangial cells. n = 3.

Additionally, we investigated the effect of curcumin and hemin on cellular autophagy in H_2_O_2_-treated and normoxic glomerular mesangial cells, respectively (Figure 6). The expression ratios of LC3 II/LC3 I were measured as markers of cellular autophagy. In the excessive oxidation setting, the LC3 expression assay indicated a significantly enhanced autophagy with curcumin (*p*<0.01) and decreased autophagy with hemin (*p*<0.001). Comparatively, we did not detect any significant effect of hemin on LC3 protein expression in the normoxic setting, similar to ZnPP. Curcumin was shown to remarkably enhance the LC3 expression in normoxic glomerular mesangial cells (*p*<0.01).

## Discussion

The regulation of multiple genes and proteins can lead to significant alterations in signaling pathways, which strongly suggests that multiple mechanisms underlie the progression of nephropathies [23], [24]. From this perspective, a novel notion of multi-pathway drugs was proposed in our study. Multi-pathway drugs are presented as distinct from multi-target drugs or drugs with a specific action. We propose that more complete therapeutic responses can be achieved by pharmacologically intervening in multiple disease-related pathways. By performing functional chemical-protein association analysis, we confirmed the real existence of multi-pathway drugs. More importantly, literature search revealed nine multi-pathway agents that have been experimentally validated as potential therapeutic drugs for nephropathies.

Despite the fact that a stringent search condition was applied (confidence score≥0.9, target numbers ≥10), the STITCH database retrieval identified more than 700 multi-target chemicals. Based on a the huge number of proteins expressed in the human body [25], this result implies a high probability that a chemical or a drug is functionally associated with more than one gene products. Targeting multiple functionally related proteins endows chemicals with the potential to correct the dysregulated cellular signaling caused by disease [26]. Despite the lack of a specific action compared to single-target drugs, multi-target drugs possess more advantages such as requiring smaller doses and having superior therapeutic effects [27]–[29]. Owing to the genetic variation in protein-coding genes, the normal function of gene products may be damaged by gene encoding errors like single gene polymorphism [30]. It is easy to understand that this may lead to treatment failure of single-target drugs [31]. In contrast, this dilemma is unlikely encountered by multi-target drugs, especially by the proposed multi-pathway drugs. Multi-pathway drugs are even less likely to be met with the development of drug resistance compared with multi-target drugs. This advantage would allow multi-pathway drugs wider applications for the patient population.

Our result indicates that the KEGG pathway “drug metabolism” was most susceptible to multi-target chemicals. This was not surprising when we consider the number of drugs that are metabolized by pathways such as the cytochrome P450 system [32]. Theoretically, with the increasing number of target proteins, a drug may be involved in more signaling pathways. However, it would not be prudent to select the most extensive protein-targeting chemicals in therapeutic drug design as there exists a correlation between the number of drug targets and the frequency of the occurrence of drug-related side effects [10]. With this in mind, we proposed the parameter PW to quantify the effectiveness of a multi-target drug on pathway intervention. For example, curcumin was assigned a PW value of 0.636. This mathematically states that curcumin can affect 0.636 signaling pathways per one target. Not surprisingly, we found that endogenous substances are generally associated with the highest number of proteins. However, the PW calculation reveals low-efficiency of these endogenous chemicals in regulating pathways in quantity. For instance, the function of 864 proteins was shown to require the involvement of calcium ions. However, the PW value for calcium was calculated to be 0.05. This value is quite low and illustrates the inefficiency of calcium in influencing a sufficient number of pathways in the human body as a whole.

By combining PW calculations and literature searches, we recognized nine multi-pathway renoprotectants, among which curcumin has been the most studied. Numerous renoprotective effects of curcumin have been noted in the literature, such as anti-inflammatory effects, anti-oxidative effects, and anti-apoptosis effects [33]–[42]; it is also known to be involved in HO-1 activity regulation [43]. These findings are in line with our result showing that curcumin is extensively involved in nephropathy-related pathways. Our demonstration of curcumin's effects on ROS content, autophagocytic activity, and apoptosis validates the multi-pathway nature of curcumin's activity.

An increased level of ROS is thought to be an essential factor for altered autophagy in cells [44]. It has been demonstrated that increased autophagy is advantageous in repairing early cell injuries [45], [46]. Consistent with a previous study [47], our results verified the direct effect of curcumin on autophagy. HO-1 is the only source of endogenous carbon monoxide (CO), and the role of HO-1 in regulating autophagy derives from its reaction product CO. CO catalyzes the oxidative breakdown of heme, which is synthesized in the mitochondria [48]. CO activates autophagy via mitochondrial ROS generation, and the mitochondria-targeting antioxidant Mito-TEMPO can abolish this role of CO [49]. Given the knowledge of the specific role of HO-1 in autophagy, we proposed that the low content of ROS found under normal conditions might explain the ineffectiveness of hemin in altering the basal autophagy level due to low abundance of heme. It is also noteworthy that HO-1 itself is a characteristic antioxidant and can reduce the external oxidative stress-induced ROS generation. Owing to the sequential association between oxidative stress and autophagy [50], it can be assumed that free radical scavenging activity by hemin-activated HO-1 may offset or even reverse the actual effect of CO in activating autophagy. This assumption was validated by the results of the present study and the findings of previous experiments performed in renal proximal tubule cells and cerebellar Purkinje cells [51], [52]. Thus, reduced autophagy should not be attributed to a protective effect of hemin against H_2_O_2_-induced cell injuries.

In conclusion, we have established a feasible method to search for multi-pathway renoprotectants. In vitro studies have revealed the advantage of multi-pathway drugs in repairing injured renal cells. Nonetheless, more in vivo studies are needed to further evaluate the usefulness of multi-pathway drugs, not only in nephropathies but also in other complex human diseases.

## Supporting Information

Table S1 **Multi-target chemicals included in this study.**
(XLS)

Table S2 **Association between chemicals and KEGG pathways.**
(XLS)

Table S3 **Nephropathy-related KEGG pathways.**
(XLS)

Table S4 **Evidence for multi-pathway renoprotectants of high pathway wideness.**
(XLS)
